# Interventional Study for Improving Health Information System in Khyber Pakhtunkhwa, Pakistan

**DOI:** 10.7759/cureus.11785

**Published:** 2020-11-30

**Authors:** Rab Nawaz, Shahzad Ali Khan, Tayyaba Khattak, Fatima Nasir, Kiran Abbas

**Affiliations:** 1 Community Medicine, Pak International Medical College, Peshawar, PAK; 2 Public Health, Health Services Academy, Islamabad, PAK; 3 Medicine, Jinnah Postgraduate Medical Centre, Karachi, PAK; 4 Medicine and Surgery, Sindh Medical College, Karachi, PAK

**Keywords:** district health information system, prism, use of information, world health organization, pakistan, khyber pakhtunkhwa, healthcare system

## Abstract

Objective

To assess the improvement in the health information system in the district Nowshera by integrating the data reporting of the Expanded Program on Immunization (EPI) and Lady Health Worker (LHW) programs in the existing system.

Methodology

The study was conducted at district Nowshera and Swabi, Pakistan between May 2015 and May 2016 for a duration of one year. The data collection instruments used in the study were adapted from the Performance of Routine Information System Management (PRISM) tool package. The study was conducted in three phases during a period of one year. The first three months were utilized for baseline assessment. The next six months were being used for implementing the integration of the EPI and LHW, and the next three months were being used for the post-intervention evaluation. Microsoft Excel software was used to enter and analyze the data. A p < 0.05 was considered as the cut-off value for significance.

Results

The results indicated that the integration of data from the EPI and LHW with that of the existing Health Information System (HIS) is possible and has the potential for improving the existing system. The least significant results were produced by the use of information, which depicts that the utilization of data in decision making or policy making is still needed to be improved. Moreover, we reported a lack of enforcement and regulation by the authorities in monitoring the feedback system in the HIS.

Conclusion

The current study revealed significant improvements in the use of information, data quality, and behavior of staff. It is essential to properly train the team on how to operate the District Health Information System (DHIS) to gain adequate and timely data on health status and determinants. Additionally, the integration would benefit in managing the data at not only the national level but at the district level too.

## Introduction

The health information system (HIS) provides reliable, authentic, and timely information on the health status of a region and aids in analyzing large amounts of data in summarized forms, which further guides the policymakers to improve upon the healthcare system [1-2]. HIS helps the policymakers to detect issues and make evidence-based decisions on health policies and programs; hence, improving the healthcare system [3].

HIS is one of the essential core elements of a healthcare system, as described by the WHO framework [4]. A reliable and trustworthy information system gives the pivot to make decisions about the structure of health organizations at any level. However, discrepancies and untimely information in the HIS system may render the system useless, further deteriorating the overall healthcare situation in any country [5].

One of the frameworks, called Performance of Routine Information System Management (PRISM), is being utilized for improving the health information system, worldwide [6]. PRISM comprises an applied system and related information assortment and scrutiny apparatuses to survey, plan, reinforce, and assess HIS. The PRISM system can be utilized for evaluating HIS execution, procedures, and its major components, specialized and social elements [7].

The structure of HIS in Pakistan is not integrated at the sub-district, district, provincial and national levels; instead, it is in the form of fragments with multiple vertical systems operating individually. The Health Management Information System (HMIS) was set up by the government of Pakistan in 1992 [8]. Later in 2004 and 2007, an improved system called the District Health Information System (DHIS) was established in collaboration with the United Nations Population Fund (UNFPA) [9]. The implementation was made sure through the National action plan. DHIS protocol was implemented throughout the country in a phase-wise process effected from 2005. However, despite the recent developments, there is still a critical need to improve the health information system in the health sector of the country in general and Khyber Pakhtunkhwa in specific. Hence, the current study aims to evaluate a modified conceptual framework, PRISM, to assess routine health information systems in two districts of Khyber Pakhtunkhwa. Additionally, the authors studied the impact of the integration of the data reporting of the Expanded Program on Immunization (EPI) and Lady Health Worker (LHW) programs with the health information system in district Nowshera of Khyber Pakhtunkhwa.

## Materials and methods

The present interventional study was conducted from June 2015 to June 2016 in two districts i.e., district Nowshera and Swabi of Khyber Pakhtunkhwa. The study was conducted in three phases. The first three months were utilized for baseline assessment of DHIS. The next six months were used for implementing the integration of the EPI and LHW program altogether in a single information system of DHIS. Finally, the post-intervention assessment was conducted.

The functioning of the Provincial District Health Information (DHIS) Cell, Districts Health Offices, and Health Facilities and the health staff involved in the process of health information systems were analyzed and recorded. Furthermore, the study recruited data to learn the impact of the integration. For evaluating the effects of integration, the two districts Nowshera and Swabi were selected. Swabi district, where the DHIS system was well-established, was kept as the control. The data collection instruments used in this study were adapted from the PRISM tool package that was modified for this study [10]. This tool package addresses the utilization of routine health information system (RHIS) data that is collected within the one-year range from the health facilities [2].

The following data collection tools (questionnaires) were adopted for assessment of the health information system in district Nowshera and district Swabi (see the Appendix):

Organizational and Behavioral Assessment Tool (OBAT)

DHIS Diagnostic Tool Health Facility Proforma: Quality of Data

DHIS Diagnostic Tool Health Facility Proforma: Use of Information

The data regarding DHIS performance, the efficacy of the managers and their subordinates, the use of DHIS data by the district office, and supervision by the district health office were assessed using the above-mentioned questionnaires. The intervention of integration was carried out in the district Nowshera while the Swabi served as a control district. The two districts share similar sociodemographics [11]. The total number of health facilities being operational in Nowshera was 33 at the time of the study conducted. Out of these 33 facilities, 13 facilities were selected randomly. Besides evaluating the technical capabilities of the district, the culture of using the health information system for assessing the progress of the health system is also evaluated. For that purpose, a set of questions were prepared according to the score of which the acceptability of using the DHIS was analyzed. The study population was categorized into two major groups, the administrative units included the district/health facilities and the second group included the employees that managed the health information systems. All the primary and secondary level health facilities in the public sector were involved in the process of integration.

After baseline evaluation, the health staff of the health information system was trained in data collection and timely reporting. Intervention (intervention in the shape of an integrated form of the process of data collection and timely reporting) was conducted in district Nowshera. After six months of the interventional approach, findings were compared with district Swabi. The process of data collection was carried out through various stakeholders. As described earlier, the PRISM package consists of three inbuilt tools: Use of information, quality of data, and Organizational & Behavioral Assessment Tool (OBAT). SPSS version 26 (IBM Corp., Armonk, NY) was used to enter and analyze data. Chi-square test and Mann-Whitney-U were used to analyze the pre- and post-intervention data. A p-value of less than 0.05 was considered statistically significant.

## Results

In the pre-intervention period, all three tools produced non-significant results. However, after intervention and integration of EPI and LHW programs, we found significant improvements in DHIS, Nowshera with respect to compilation of the reports containing the DHIS data and in receiving the feedback report from the DHO office. In displaying the information about the health of the mother, child, and disease surveillance again we got significant results. After the integration of DHIS in the Nowshera district, the significant results could also be seen in the decision-making process while using the information obtained from the Nowshera’s facilities. A significant change was also marked in the behavior of the district health officer as he started visiting the facilities in three or six months. The record-keeping, the maintenance of the record, and using that record in making the decision and setting the health targets showed a significant response (Table 1).

**Table 1:** Use of Information (Post Intervention)

Query		Nowshera (n%)		Swabi (n%)		p-value
Does this facility compile DHIS Data?	Yes	24	80.00%	12	40.00%	0.002
	No	6	20.00%	18	60.00%	
Does the facility compile any report containing DHIS information?	Yes	20	66.67%	8	26.67%	0.002
	No	10	33.33%	22	73.33%	
Did the facility receive any feedback from the district office on their performance for the last three months?	Yes	6	20.00%	0	0.00%	0.010
	No	24	80.00%	30	100.00%	
Display of Information						
Does the district office display the following data:						
Mother Health	Yes	18	60.00%	0	0.00%	0.000
	No	12	40.00%	30	100.00%	
Child Health	Yes	16	53.33%	3	10.00%	0.000
	No	14	46.67%	27	90.00%	
Facility Utilization	Yes	13	43.33%	2	6.67%	0.001
	No	17	56.67%	28	93.33%	
Disease Surveillance	Yes	22	73.33%	5	16.67%	0.000
	No	8	26.67%	25	83.33%	
Does the office have a map of the catchment area?	Yes	10	33.33%	2	6.67%	0.010
	No	20	66.67%	28	93.33%	
The office displays a summary of demographic information:	Yes	12	40.00%	1	3.33%	0.001
	No	18	60.00%	29	96.67%	
Any feedback (quarterly, yearly) report on DHIS data	Yes	10	33.33%	0	0.00%	0.001
	No	20	66.67%	30	100.00%	
If yes, what kinds of decisions are made in reports of DHIS data/information?						
Review strategy by examining actual performance on a month to month comparisons	Yes	8	80.00%	-	-	-
	No	2	20.00%	-	-	-
Review facility personnel responsibilities by examining service performance	Yes	3	30.00%	-	-	-
	No	7	70.00%	-	-	-
Mobilization/shifting of resources based on the comparison by services	Yes	2	20.00%	-	-	-
	No	8	80.00%	-	-	-
Advocacy for more resources by comparing performance by targets	Yes	6	60.00%	-	-	-
	No	4	40.00%	-	-	-
Discussion and Decision on DHIS information						
Does the facility have routine meetings for reviewing managerial matters?	Yes	16	53.33%	4	13.33%	0.001
	No	14	46.67%	26	86.67%	
How frequently is the meeting supposed to take place?		3		3		
							How many times did the meeting take place during the last three months?		2		1		
Is an official record of management meetings maintained?	Yes	13	81.25%	2	50.00%	0.004							
	No	3	18.75%	2	50.00%	
If yes, please check the meeting records for the last three months:						
Management of DHIS, such as data quality, reporting, or timeliness of reporting	Yes	13	100.00%	2	100.00%	
	No	0	0.00%	0	0.00%	
Discussion on DHIS findings such as disease data, or service coverage, medicine	Yes	13	100.00%	2	100.00%	
	No	0	0.00%	0	0.00%	
Have they made any decisions based on the above discussions?	Yes	6	46.15%	0	0.00%	0.003
	No	7	53.85%	2	100.00%	
Any follow-up action taken on the decisions made during the previous meetings?	Yes	2	33.33%	0	0.00%	0.102
	No	4	66.67%	2	100.00%	
Any DHIS related issues/problems referred to at the provincial level for actions?	Yes	0	0.00%	0	0.00%	
	No	6	100.00%	2	100.00%	
Use of Information by the District Office						
Facility received annual/monthly planned targets based on DHIS information?	Yes	13	43.33%	0	0.00%	0.000
	No	17	56.67%	30	100.00%	
Did records of the facility of the last three months show that district directives?	Yes	12	40.00%	0	0.00%	0.000
	No	18	60.00%	30	100.00%	
Did the facility receive the district DHIS office newsletter/report in the last three months?	Yes	0	0.00%	0	0.00%	
	No	30	100.00%	30	100.00%	
Does any documentation exist to show us information?	Yes	0	0.00%	0	0.00%	
	No	30	100.00%	30	100.00%	
Did the person in charge of the facility participate in meetings at the district level to discuss DHIS performance for the last three months?	Yes	4	13.33%	0	0.00%	0.038
	No	26	86.67%	30	100.00%	
Examples of how the facility uses DHIS information for health system management	Yes	16	53.33%	5	16.67%	0.003
	No	14	46.67%	25	83.33%	
Facility received annual/monthly planned targets based on DHIS information	Yes	4	13.33%	0	0.00%	0.038
	No	26	86.67%	30	100.00%	
Supervision by the district health office						
Did the district supervisor visit your facility during the last three months?	Yes	6	20.00%	0	0.00%	0.010
	No	24	80.00%	30	100.00%	
Did you observe the supervisor having a checklist to assess the data quality?	Yes	7	23.33%	0	0.00%	0.005
	No	23	76.67%	30	100.00%	
Did the supervisor check the data quality?	Yes	2	6.67%	0	0.00%	0.150
	No	28	93.33%	30	100.00%	
Did the district supervisor discuss the performance of health facilities based on DHIS information when he visited your facility?	Yes	6	20.00%	0	0.00%	0.010
	No	24	80.00%	30	100.00%	
Did the supervisor help you make a decision based on DHIS information?	Yes	0	0.00%	0	0.00%	
	No	30	100.00%	30	100.00%	
Did the supervisor send a report/feedback/note on the last two supervisory visits?	Yes	2	6.67%	0	0.00%	0.150
	No	28	93.33%	30	100.00%	

Upon assessing the OBAT, the staff was more punctual and set targets regularly for them to achieve post-intervention. Staff practiced saying no to any decision which was not supported by the evidence or facts and the culture of accepting the mistakes and rectifying it later flourished inside the facilities. The Nowshera staff were more capable and self-efficient in interpreting the data and on the basis of data made graphs and charts to depict the monthly progress of the facility compared to the control district, i.e. Swabi, in the post-intervention phase (Table 2).

**Table 2:** Organizational and Behavioral Assessment Tool (OBAT) (Post Intervention)

Item	Nowshera	Swabi	p-value
	Mean Score		Mann-Whitney-U
In the health department, decisions are based on:			
Personal liking	4.83	5.7	0.000
Superiors’ directives	6.7	5.6	0.000
Evidence/facts	2.17	2.03	0.502
Political interference	5.67	5.6	1.000
Comparing data with strategic health objectives	1.67	2	0.112
Health needs	1.87	1.73	0.586
Considering costs	3.43	3.23	0.469
In the health department, superiors;			
Seek feedback from concerned persons	1.57	1.63	0.882
Emphasize data quality in monthly reports	2.03	1.87	0.428
Discuss conflicts openly to resolve them	1.97	2.63	0.007
Seek feedback from concerned community	2.03	2.43	0.157
Use HMIS data for setting targets and monitoring	2.1	1.87	0.230
Check data quality at the facility and higher level regularly	2	1.83	0.385
Provide regular feedback to their staff through regular report based on evidence	2.4	2.63	0.271
Report on data accuracy regularly	1.93	2.4	0.141
In the health department, staff			
Are punctual	1.7	2.83	0.000
Document their activities and keep records	2.37	1.63	0.001
Feel committed to improving the health status of the target population	2.07	1.87	0.428
Set appropriate and doable target for their performance	1.7	2.63	0.000
Feel guilty for not accomplishing	2.2	2.43	0.403
Are rewarded for good work	2	1.87	0.387
Use HMIS data for day to day management of the facility and district	1.77	1.83	0.745
Display data for monitoring their set target	1.8	2.63	0.004
Can gather data to find the root cause(s) of the problem	1.8	2.4	0.044
Can develop appropriate criteria for selecting interventions for a given problem	2.33	2.63	0.359
Can develop appropriate outcomes for a particular intervention	2.07	2.43	0.189
Can evaluate whether the targets or outcomes have been achieved	1.93	1.87	0.538
Are empowered to make decisions	2.6	1.83	0.003
Able to say no to superiors and colleagues for demands/decisions not supported by evidence	1	2.63	0.000
Are made accountable for poor performance	5.97	5.53	0.056
Use HMIS data for community education and mobilization	2.3	1	0.000
Admit mistakes for taking corrective actions	2.47	1	0.000
Personal			
Collecting information which is not used for decision making discourages me	1.93	2.63	0.005
Collecting information makes me feel bored	5.97	5.83	0.637
Collecting information is meaningful for me	2.2	1.4	0.001
Collecting information gives me the feeling that data is needed for monitoring facility performance	2.2	2.43	0.370
Collecting information gives me the feeling that it is forced on me	1.9	1.63	0.213
Collecting information is appreciated by Co-workers and superiors	2	1.67	0.114
Self-Efficacy			
I can calculate percentages/rates correctly	2.33	0	0.005
I can plot data by months or years	1.67	0	0.021
I can compute trend from bar charts	1.67	0	0.021
I can explain findings & their implications	1.67	0	0.021
I can use data for identifying gaps and setting targets	1	0	0.780
I can use data for making various types of decisions and providing feedback	1.67	0	0.210

The quality of data after integration improved as many queries from the PRISM tool package showed significant differences. The process of reporting and meeting the deadlines for the submission of these reports improved post-intervention (Table 3).

**Table 3:** Quality of Data (Post Intervention)

Query		Nowshera		Swabi		p-value
Keeping Record	Yes	12	40.00%	12	40.00%	
	No	0	0.00%	0	0.00%	
Number of facilities actually reporting	Yes	24	80.00%	14	46.67%	0.007
	No	6	20.00%	16	53.33%	
Deadline for submission of monthly report	Yes	24	80.00%	14	46.67%	0.007
	No	6	20.00%	16	53.33%	
A Reporting Month A	Before the deadline	24	80.00%	13	43.33%	0.003
	After the deadline	6	20.00%	17	56.67%	
Availability of person for collection of monthly report	Yes	24	80.00%	18	60.00%	0.091
	No	6	20.00%	12	40.00%	
Data Accuracy	Yes	14	46.67%	12	40.00%	0.602
	No	16	53.33%	18	60.00%	
Indicators for Each Facility Catchment Area	Yes	10	33.33%	7	23.33%	0.390
	No	20	66.67%	23	76.67%	
Comparison among Facilities	Yes	5	16.67%	6	20.00%	0.739
	No	25	83.33%	24	80.00%	
Comparison among Type of services	Yes	5	16.67%	7	23.33%	0.519
	No	25	83.33%	23	76.67%	
Is monthly report form complex and difficult to follow	Yes	9	30.00%	20	66.67%	0.004
	No	21	70.00%	10	33.33%	
Do you find that IT is easy to manage?	Yes	19	63.33%	10	33.33%	0.020
	No	11	36.67%	20	66.67%	
DHIS has information that is spread over in different information system	Yes	19	63.33%	16	53.33%	0.432
	No	11	36.67%	14	46.67%	
(LAN) exist to provide access to information to all district managers	Yes	11	36.67%	9	30.00%	0.584
	No	19	63.33%	21	70.00%	

## Discussion

In Pakistan, adequate and timely information from the District Health Information System (DHIS) is hindered by lack of facility to record the data systematically, lack of feedback system, lack of utilization of knowledge in taking decisions and disease surveillance, inefficient management, power politics, and the incapability of the staff to adapt to the modern system [11-13]. The current study assessed routine health information systems in two districts of Khyber Pakhtunkhwa using a modified conceptual framework, PRISM.

Considering the findings of the current and the previous studies, we can conclude that one of the obstacles in improving the HIS is the poor management of the existing resources. The reasons behind this include poor management of data, low quality of data due to data duplication, selection of data without taking the technicalities into account, lack of proper channel for timely and updated transmission of data to the national level and lack of coordinated efforts to address the problems of the periphery to the district and then to the national level respectively. The health workers do not have access to the proper and standardized training through which they could develop an understanding of the procedure for the collection and processing of the data [14]. Furthermore, there is a lack of motivation and financial incentives for the health services workers due to which they tend to lose interest in their work, and chances of errors increase. The lack of a feedback system is another reason behind this low quality of data [13-15].

The current study used a modified PRISM tool to highlight the main issues concerning the DHIS. It was noted that there was no proper management system to ensure the timely transmission of the data from district to provincial and from provincial to the national level. This resulted in outdated, low-quality data, which further affected the decisions made without any sound evidence. Additionally, in many facilities computers were not being used as the staff was not skilled in operating a computer. The utilization of some straightforward and sophisticated programs like GIs, and EPlINFO was also not very popular in the health sector in Pakistan. Similar findings were found in previous studies [16-17].

The research has shown that we lack the management along with the resources. The integration of EPI & LHW data with the data of HIS in the DHIS software is a positive initiative for upgrading the health information system from the district level. Still, this integration needs a strict follow-up procedure that ensures that the system keeps on working the way they are supposed to do. The HMIS operating in the facilities could be used as the most powerful tool for planning and managing health services. To establish a system that could prove to be efficient enough to respond to the needs of making a decision based upon the information from the healthcare delivery system, we need to have a vast health information system that should have the ability to process all over the country in terms of infrastructure and networking. On a general assessment of the existing health system, it was found out that the overall design is very feeble, the data collection system is not that organized, and information is disseminated in fragments. Because of this situation, efforts should be arranged where the prime focus should be on the organization of data, utilization of the data, and dissemination of the data to the respective stakeholder.

## Conclusions

The current study used the PRISM framework to highlight the main challenges in improving DHIS in Khyber Pakhtunkhwa. It was found that there was no proper management system that ensured the timely transmission of the data from district to provincial and from provincial to the national level. This resulted in outdated, low-quality data which further affected the decisions made without any sound evidence. Additionally, in many facilities computers were not being used as the staff was not skilled in operating a computer. After the integration of EPI and LHW programs, significant improvements in the use of information, data quality, and behavior of staff after the intervention were observed. In short, it is important to properly train the staff on how to operate DHIS in order to gain adequate and timely data on health status and determinants. The integration would benefit in managing the data at not only the national level but at the district level too.

## Appendices

**Table 4:** DHIS Diagnostic Tool Health Facility Proforma: Quality of Data

Serial #				Question				Response						
Data Recording														
FQ 1	Copy of DHIS monthly reports sent to the district office?						1. Yes				0. No. If no, go to FQ5			
FQ 2	# of DHIS monthly reports kept at the facility for the last 12 months													
FQ 3	Does this facility keep an outpatient register?						1. Yes				0. No. If no, go to FQ5			
Data Accuracy Check														
FQ 4	Find the following information for the two months in the outpatient register													
	Item		a. Month (specify)			b. Month (specify)								
			# from register	# from report		# from register				# from report				
4A														
4B														
4C														
4D														
FQ 5	Did you receive a directive from the DHO/DHIS Coordinator to:													
5A. Check the data accuracy at least once in three months?									1. Yes				0. No	
5B. Fill the monthly report form completely									1. Yes				0. No	
5C. Submit report by due date									1. Yes				0. No	
FQ 6	Did you receive a directive from the District office that there will be consequences:													
6A. If you do not check the data accuracy									1. Yes				0. No	
6B. If you do not fill the monthly reporting form completely									1. Yes				0. No	
6C. If you do not submit the monthly report by the declared deadline									1. Yes				0. No	
Data Completeness														
FQ 7	# of data items in the DHIS monthly report that the facility needs to report?													
FQ 8	# of data items left blank without indicating “0” in the last month's report.													
Data Transmission/Data Processing & Analysis														
FQ 9	Do data processing procedures exist?							1. Yes					0. No	
FQ 10	Does the facility produce the following?													
FQ A	Indicator of facility catchment area							1. Yes					0. No	
FQ B	Comparisons with district/national targets							1. Yes,					0. No	
FQ C	Comparisons among types of services coverage							1. Yes					0. No	
FQ D	Comparisons of data over time (monitoring over time)							1. Yes					0. No	
FQ 11	Does the procedure manual for data collection/definitions exist?							1. Yes					0. No	

**Table 5:** Self-efficacy Scale

Please rate your confidence in percentages that you can accomplish the HMIS activities. Rate your confidence for each situation with a percentage from the following scale:												
Serial #	Item	Scores										
SE2.	I can calculate percentages/rates correctly	0	10	20	30	40	50	60	70	80	90	100
SE3.	I can plot data by months or years	0	10	20	30	40	50	60	70	80	90	100
SE4.	I can compute trend from bar charts	0	10	20	30	40	50	60	70	80	90	100
SE5.	I can explain findings & their implications	0	10	20	30	40	50	60	70	80	90	100
SE6.	I can use data for identifying gaps and setting targets	0	10	20	30	40	50	60	70	80	90	100
SE7.	I can use data for making various types of decisions and providing feedback	0	10	20	30	40	50	60	70	80	90	100

**Table 6:** Organizational and Behavioral Assessment Tool (OBAT) A (Strongly disagree), B (Somewhat Disagree), C (Disagree), D (Neither Agree nor disagree), E (Agree), F (Somewhat agree), G (Strongly agree).

Serial #	Question	A	B	C	D	E	F	G
In the health department, decisions are based on								
D1.	Personal liking							
D2.	Superiors’ directives							
D3.	Evidence/facts							
D4.	Political interference							
D5.	Comparing data with strategic health objectives							
D6.	Health needs							
D7.	Considering costs							
In the health department, superiors								
S1.	Seek feedback from concerned persons							
S2.	Emphasize data quality in monthly reports							
S3.	Discuss conflicts openly to resolve them							
S4.	Seek feedback from concerned community							
S5.	Use HMIS data for setting targets and monitoring							
S6.	Check data quality at the facility and higher level regularly							
S7.	Provide regular feedback to their staff through regular report based on evidence							
S8.	Report on data accuracy regularly							
In health department, staff								
P1.	Are punctual							
P2.	Document their activities and keep records							
P3.	Feel committed in improving health status of the target population							
P4.	Set appropriate and doable target of their performance							
P5.	Feel guilty for not accomplishing the set target/performance							
P6.	Are rewarded for good work							
In health department, staff								
P7.	Use HMIS data for day to day management of the facility and district							
P8.	Display data for monitoring their set target							
P9.	Can gather data to find the root cause(s) of the problem							
P10.	Can develop appropriate criteria for selecting interventions for a given problem							
P11.	Can develop appropriate outcomes for a particular intervention							
P12.	Can evaluate whether the targets or outcomes have been achieved							
P13.	Are empowered to make decisions							
P14.	Able to say no to superiors and colleagues for demands/decisions not supported by evidence							
P15.	Are made accountable for poor performance							
P16.	Use HMIS data for community education and mobilization							
P17.	Admit mistakes for taking corrective actions							
Personal								
BC1.	Collecting information which is not used for decision making discourages me							
BC2.	Collecting information makes me feel bored							
BC3.	Collecting information is meaningful for me							
BC4.	Collecting information gives me the feeling that data is needed for monitoring facility performance							
BC5.	Collecting information gives me the feeling that it is forced on me							
BC6.	Collecting information is appreciated by Co-workers and superiors							
